# Oligosaccharides Derived from Tramesan: Their Structure and Activity on Mycotoxin Inhibition in *Aspergillus flavus* and *Aspergillus carbonarius*

**DOI:** 10.3390/biom11020243

**Published:** 2021-02-08

**Authors:** Jelena Loncar, Barbara Bellich, Alessia Parroni, Massimo Reverberi, Roberto Rizzo, Slaven Zjalić, Paola Cescutti

**Affiliations:** 1 Department of Ecology, Aquaculture and Agriculture, University of Zadar, Mihovila Pavlinovića 1, 23000 Zadar, Croatia; jloncar@unizd.hr; 2 Department of Environmental Biology, Sapienza University of Rome, P.le Aldo Moro 5, 00185 Rome, Italy; alessia.parroni1@gmail.com (A.P.); massimo.reverberi@uniroma1.it (M.R.); 3 Department of Life Sciences, University of Trieste, Via Licio Giorgieri 1, Bdg. C11, 34127 Trieste, Italy; bbellich@units.it (B.B.); rizzor@units.it (R.R.); pcescutti@units.it (P.C.)

**Keywords:** mycotoxin, Tramesan, biocontrol, aflatoxins, ochratoxin A, oligosaccharides

## Abstract

Food and feed safety are of paramount relevance in everyday life. The awareness that different chemicals, e.g., those largely used in agriculture, could present both environmental problems and health hazards, has led to a large limitation of their use. Chemicals were also the main tool in a control of fungal pathogens and their secondary metabolites, mycotoxins. There is a drive to develop more environmentally friendly, “green”, approaches to control mycotoxin contamination of foodstuffs. Different mushroom metabolites showed the potential to act as control agents against mycotoxin production. The use of a polysaccharide, Tramesan, extracted from the basidiomycete *Trametes versicolor*, for controlling biosynthesis of aflatoxin B1 and ochratoxin A, has been previously discussed. In this study, oligosaccharides obtained from Tramesan were evaluated. The purified exopolysaccharide of *T. versicolor* was partially hydrolyzed and separated by chromatography into fractions from disaccharides to heptasaccharides. Each fraction was individually tested for mycotoxin inhibition in *A. flavus* and *A. carbonarius*. Fragments smaller than seven units showed no significant effect on mycotoxin inhibition; heptasaccharides showed inhibitory activity of up to 90% in both fungi. These results indicated that these oligosaccharides could be used as natural alternatives to crop protection chemicals for controlling these two mycotoxins.

## 1. Introduction

Mycotoxins are fungal secondary metabolites that represent a serious threat to the health of both animals and humans [1]. Food and feed contamination by mycotoxins poses major concerns for public health and welfare as dietary exposure may cause disorders, dysfunctions, and alterations of physiological states in both humans and animals [2]. Ripening staple crops are all exposed to phyllosphere fungi, some of which are able to infect the crops resulting in mycotoxin contamination [3]. Many countries have strict regulations to minimize the exposure of humans and animals to the key mycotoxins [4,5]. However, the allowed levels of contamination are not harmonized among countries, and this may cause trade frictions at the global level [6]. The presence of aflatoxins (AFs) in food and feed can have a variety of toxic effects, e.g., hemorrhages, hepatotoxicity, nephrotoxicity, neurotoxicity, estrogenicity, teratogenicity, immunosuppressive problems, mutagenicity, and carcinogenicity [7,8,9,10,11,12]. It is known from the literature that aflatoxins are capable of inducing liver tumors and immune-depression in humans [13,14]. According to the International Agency for Research on Cancer (IARC), aflatoxins are classified as carcinogenic to humans (Group 1A). For this reason, aflatoxins are strictly limited by European laws [5]. Moreover, Ochratoxin A (OTA) represents a severe health hazard for humans and animals [15]. OTA contamination occurs in a variety of food and feed, such as coffee beans, spices, meat, cheese products, and wine [16] Moreover, studies have shown that OTA has nephrotoxic, hepatotoxic, embryotoxic, teratogenic, neurotoxic, immunotoxic, genotoxic, and carcinogenic effect on humans and animals [17]. Therefore, the IARC evaluated the carcinogenic potential of OTA as a possible human carcinogen (Group 2B), based on a large body of evidence of carcinogenicity detected in several animal studies [18]. The common use of chemicals (pesticides and fungicides) for inhibition of fungal growth and to control mycotoxin synthesis have shown hazardous side effects, such as strong environmental pollution with severe damage for the human and animal health and selection of resistant strains. The raising awareness that different chemicals could cause both environmental problems and health hazard, led to a large limitation of their use in agriculture. The European Community (EC) has banned about 50% of the chemicals commonly used in crop production since 2014 [19]. In addition, nowadays the EC policy is driving research to investigate more environmentally friendly “green” approaches. This has increased the research on the development of biocontrol strategies as well as the use of natural plant extracts to control and mitigate the presence of mycotoxins in food and feed [20,21,22,23,24]. One of the promising tools for mycotoxin (particularly aflatoxins) control, with lower environmental impact, are the metabolites of higher mushrooms. It has been demonstrated that among the factors affecting mycotoxin biosynthesis in *A. flavus* and *A. parasiticus*, a critical and pivotal role is played by intracellular and environmental oxidative stress. The close relationship between endogenous oxidative state and AF biosynthesis, and the direct relation between increased levels of both reactive oxygen species (ROS) and aflatoxin biosynthesis in *A. parasiticus* has been demonstrated [1,14,25,26]. Mushrooms contain large amounts of mycochemicals that possess antioxidant properties and have a strong free radical scavenging ability [27]. Many, if not all, higher basidiomycete mushrooms contain biologically active polysaccharides in fruit bodies, cultured mycelium, and cultured broth [28,29,30], and they are generally considered to be the main contributors to the antioxidant activity [31]. Some of these polysaccharides are described as biological response modifiers (BRM); these include compounds with specific biological functions: antibiotics (e.g., plectasin), immune system stimulators (e.g., lentinan), antitumor agents (e.g., polysaccharide-K known also as krestin or PSK) and hypolipidemic agents (e.g., lovastatin), inter alia [31]. The possibility of using mushroom polysaccharides as a mean to control aflatoxins synthesis has been widely demonstrated [32,33,34,35]. Probably, the most studied are the polysaccharides produced by the mushroom *Trametes versicolor*. It has been demonstrated that *T. versicolor* lyophilized culture media and mycelial extracts showed long-lasting ability to inhibit the synthesis of aflatoxins both in vitro and in vivo [30,36]. The active component of the extracts was found to be a polysaccharide, which was isolated, part of its primary structure determined and registered as Tramesan. Tramesan^©^ is a branched fucose-containing polysaccharide of about 23 kDa with a “repetitive” scheme of monosaccharide sequence in the linear (α-1,6-Gal)n backbone as well as in the lateral chains α-Man-(1→2)-(α-Man)_n_-(1→3)-Fuc [31]. Tramesan^©^ can act as a pro antioxidant in different organisms. By enhancing the “natural” antioxidant defenses of the “hosts”, Tramesan^©^ could represent a useful tool for controlling the synthesis of several mycotoxins simultaneously [14,32,33,34,35,36]. As demonstrated by [1], Tramesan^©^ can elicit an antioxidant response, probably by acting on gene expression. It was suggested that Tramesan^©^ could be recognized by specific receptors that, in turn, activate pathways leading to an antioxidant response (e.g., ApYapA): Tramesan^©^ could act as ligand for a still unknown inter-kingdom conserved receptor able to control antioxidant responses [37,38]. The objectives of this study were to evaluate the smallest active component of Tramesan^©^ for inhibiting aflatoxin B1 and ochratoxin A biosynthesis. This information could be important to better understand and clarify the mechanism of action of Tramesan^©^ (and possibly other mushroom polysaccharides) on mycotoxin synthesis, as well as to set up the procedures for a production of a novel—natural—anti-mycotoxigenic compound. Therefore, Tramesan^©^ was hydrolyzed and the obtained oligosaccharides were separated, structurally characterized, and individually tested for their mycotoxin inhibition activity, with a focus on AFB1 and OTA control.

## 2. Materials and Methods

### 2.1. Fungal Strain and Growth Conditions

*T. versicolor* used in this study was registered at CABI Biosciences (Egham, UK) and deposited in the culture collection of Department of Environmental Biology of Sapienza University of Rome as ITEM 117. *T. versicolor* strain 117 was grown for 7 days in potato dextrose broth (PDB, HiMedia, Mumbai, India), and incubated at 25 °C under shaken conditions (100 rpm). The liquid culture was homogenized, in sterile conditions, in Waring blender 8012. After homogenization, an aliquot (5% *v*/*v*) of the fungal culture was inoculated in sterile conditions in 500 mL of PDB in 1L-Erlenmeyer flasks and incubated for 14 days at 25 °C under rotary shaken conditions (100 rpm). The fungal biomass was then separated from the culture filtrates by subsequent filtration with different size filters (Whatman, Maidstone, UK), to eliminate all of the mycelia. Mycelia-purified culture filtrate was evaporated under reduced pressure by rotary evaporation (IKA, RV 10, basic). 1 L of culture filtrate was concentrated to a volume of 50 mL, lyophilized, and utilized for subsequent analyses. The isolates were kept on potato dextrose agar (HiMedia, Mumbai, India) at 4 °C and the cultures were sub-cultured every 30 days. 

### 2.2. Purification of Polysaccharides Produced by T. versicolor

Purification of Tramesan^©^ was performed as described in [32]. The lyophilized *T. versicolor* culture filtrate (1 g) was dissolved in 30 mL of ultrapure H_2_O and filtered to separate the insoluble components from the soluble ones. The solution was cooled at 4 °C and precipitated with 4 volumes of cold absolute ethanol. The precipitate was recovered by centrifugation at 2000× *g* for 20 min at 4 °C. The recovered pellet was re-suspended in 4 mL of ultrapure H_2_O and 4 mL of 20 mM phosphate buffer at pH 7.5 to achieve the optimal conditions of ionic strength and pH for pronase E (Merck KGaA, Darmstadt, Germany) activity. The proteolysis was carried out at 37 °C for 16 h. The sample was centrifuged (2000× *g*/40 min at 4 °C) and the supernatant was collected, dialyzed (10 kDa membrane cut off) and recovered by lyophilization.

### 2.3. Tramesan^©^ Oligosaccharide Production and Characterization of Tramesan^©^ Oligosaccharides

After deacetylation with 0.01 M NaOH at room temperature for 5 h under N_2_ flow, the polysaccharide was subjected to low pressure size exclusion chromatography (SEC) on a Sephacryl S-300 column (Merck KGaA, Darmstadt, Germany) to check the polymer homogeneity and further purify it from possible contaminants (fractionation domain: 1–400 kDa for dextrans; gel bed volume: 1.6 id × 90 cm), using 50 mM NaNO_3_ as eluent at a flow rate of 6 mL/h. The sample was separated in three loads; 1 = 50 mg, 2 = 43 mg, 3 = 40 mg. For the first loading, the sample was dissolved in 1.9 mL of eluent and centrifuged 10 min at 30,000× g and subsequently it was applied on the column. Fractions of 2 mL were collected at 20 min intervals. The same procedure was repeated with the remaining 2 parts of the sample. Elution was monitored using a refractive index detector (K-2301 KNAUER Wissen-schaftliche Geräte GmbH, Berlin, Germany), connected to a paper recorder and interfaced with a computer via PicoLog software (Pico Technology, St. Neots, UK).

The polysaccharide fractions obtained from SEC were subjected to mild acid treatment: 38.6 mg of polymer were dissolved in H_2_O at a concentration of 2 mg/mL and preheated at 100 °C for 15 min. Trifluoroacetic acid (TFA) 2 M was added to obtain a final TFA concentration of 0.5 M and the sample was incubated for 2 h at 100 °C. The hydrolysate was rotovaporated to dryness under reduced pressure at 45 °C to eliminate residual TFA, taken to pH = 7.2, rotovaporated to dryness again, dissolved in 50 mM NaNO_3_, centrifuged and separated by size exclusion chromatography on a Bio Gel P2 column (Bio-Rad Laboratories s.r.l., Milan, Italy) (fractionation domain: 100 ± 1800 Da; gel bed volume: 1.6 cm i. d. × 90 cm), using 50 mM NaNO_3_ as eluent at a flow rate of 6.4 mL/h. Fractions were collected at 15 min intervals and those belonging to the same peak were pooled together and desalted on a Bioline MPLC glass column (KNAUER Wissenschaftliche Geräte GmbH, Berlin, Germany) equipped with a Superdex G30 column, previously equilibrated in H_2_O. The purified oligosaccharides were lyophilized and subjected to nuclear magnetic resonance (NMR) spectroscopy and electrospray ionization-mass spectrometry (ESI-MS) analysis.

### 2.4. NMR Spectroscopy

The polysaccharide and purified oligosaccharides were analyzed by NMR spectroscopy. The samples were exchanged twice with 99.9% D_2_O by lyophilization and then dissolved in 0.6 mL of 99.96% D_2_O. Spectra were recorded on a 500 MHz VARIAN spectrometer (Varian Inc., Palo Alto, CA, USA) operating at 50 °C for the polymer and 25 °C for oligosaccharides solutions. Two-dimensional (2D) experiments were performed using standard VARIAN pulse sequences and pulsed field gradients for coherence selection when appropriate. Standard parameters were used for 2D NMR experiments. Chemical shifts are expressed in ppm using acetone as internal reference (2.225 ppm for ^1^H and 31.07 ppm for ^13^C). NMR spectra were processed using MestreNova software (Mestrelab Research, S.L., Santiago de Compostela, Spain).

### 2.5. ESI Mass Spectrometry

A suitable amount of oligosaccharides was dissolved in 50% aqueous methanol, 11 mM NH_4_OAc and subjected to ESI-MS experiments using a Bruker Esquire 4000 (Bruker Daltonics, Fremont, CA, USA) ion trap mass spectrometer connected to a syringe pump for the injection of the samples. The instrument was calibrated using a tune mixture provided by Bruker. Samples were injected at a flow rate of 180 μL/h and detection was performed in the positive ion mode.

### 2.6. Inhibition of Aflatoxin B1 and OTA Biosynthesis in A. flavus 3357 and A. carbonarius by Tramesan^©^ Oligosaccharide Fractions

*A. flavus* (Speare) NRRL 3357, a high AFB1 producer and *A. carbonarius* producer of OTA, were grown on PDA (HiMedia, Mumbai, India) at 30 °C for 7 days in dark conditions. A total of 90 μL of double concentrated potato dextrose broth (48 g/L) (HiMedia, Mumbai, India), 100 μL of mixture of the different oligosaccharides in sterile water), and 10 μL of conidial suspension in sterilized distilled water (1000 con/mL), were used for the experiment. The assay was performed in the presence or absence of Tramesan^©^ oligos in the concentration of 100 μM and 200 μM solutions dissolved in water, using 96-wells microplates. The cultures were incubated at 30 °C for 3 days. The assay allowed testing all of the fractions in minimal amounts, and generating hundreds of replications in a very short time (aflatoxin microtiter-based bioassay). Different cultures were independently filtered with 0.22 μm Millipore filters (Burlington, MA, USA). The extraction of AFB1 and OTA was performed with chloroform/methanol (2:1, *v*/*v*), using 5 µL of Quercetin (purity ≥ 95% Sigma-Aldrich, Merck KGaA, Darmstadt, Germany), 100 µM as recovery standard, as previously reported [38]. The mixture was vortexed for 1 min, centrifuged and then the lower phase was collected. The extraction was repeated three times and the samples concentrated under a N_2_ stream; AFB1 was re-dissolved in 50 μL of acetonitrile/water/acetic acid (20:79:1 *v*/*v*) whereas OTA in 50 μL Methanol and quantified by triple quad LC/MS 6420 (Agilent, Santa Clara, CA, USA). The amount of AFB1 was evaluated by using an ISTD-normalized method in MassHunter workstation software (Agilent, Santa Clara, CA, USA), quantitative analysis version B.07.00. Aflatoxin B1-13C-d3 (Clearsynth, Mumbai, India) at 2 μM final concentration was used as ISTD. AFB_1_ and OTA amounts were expressed in part per billions (ppb).

## 3. Results

### 3.1. Production and Purification of Oligosaccharides from Tramesan^©^

**Figure 1:**
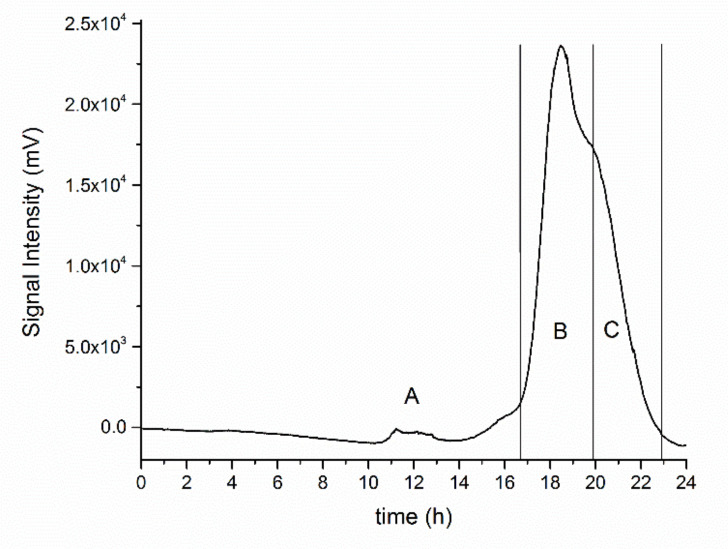
Elution profile on a Sephacryl S-300 column of the polysaccharide obtained from the culture filtrate of *T. versicolor*: bars indicate fractions, which were pooled together to give the sample polysaccharide (PLS)-B and PLS-C.

The polysaccharide (PLS) sample obtained from *T. versicolor* culture filtrate was purified according to the procedure described in the Methods section and subjected to size exclusion chromatography (SEC) to check for its homogeneity. The obtained chromatogram (Figure 1) showed an early eluting low intensity peak (A), which was disregarded, and one main peak (B) accompanied by a shoulder (C): collected test tubes were pooled as reported in Figure 1 to give two PLS fractions which were named PLS-B and PLS-C.

**Figure 2:**
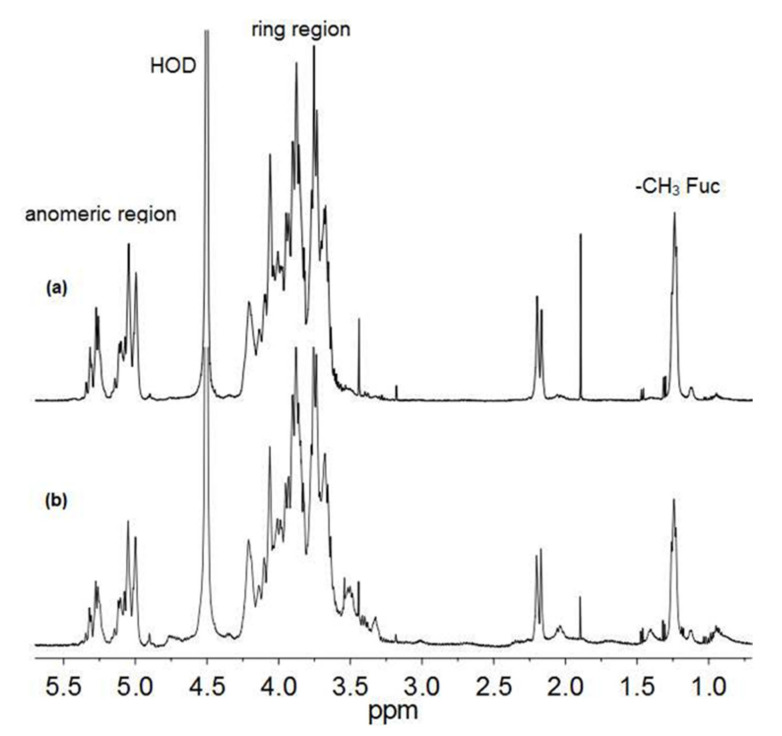
^1^H NMR spectra of PLS-B (**a**) and PLS-C (**b**) samples obtained from *T. versicolor* culture filtrate after size exclusion chromatography (SEC) separation. Spectra were recorded at 50 °C on a 500 MHz spectrometer. Main resonances are indicated.

Part of PLS-B and PLS-C were dialyzed against H_2_O, lyophilized, and exchanged with D_2_O for the subsequent ^1^H NMR analysis. The ^1^H NMR spectra of the PLS-B and PLS-C (Figure 2) indicated that the two polysaccharides are structurally very similar, with PLS-B being purer than PLS-C, and, therefore, they differ slightly for their molecular mass. Moreover, since the spectra are very similar to that already reported in a previous publication [32], the signal at 1.24 ppm was assigned to C-6 methyl group of fucose.

**Figure 3:**
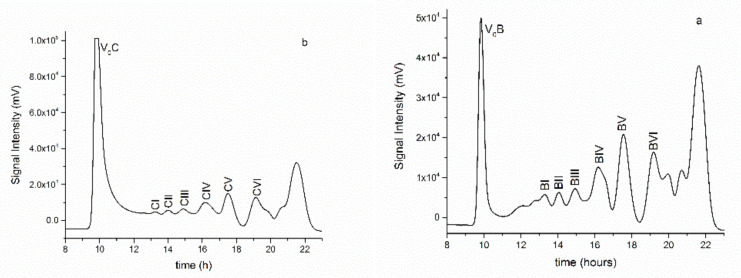
Chromatograms of the hydrolysate from PLS-B (**a**) and PLS-C (**b**) Vo indicates the excluded volumes; peaks were labeled BI–BVI and CI–CVI in order of increasing elution time.

After having verified the purity of the Tramesan^©^ fractions PLS-B and PLS-C, they were hydrolyzed using mild acid conditions in order to obtain oligosaccharides of defined size, but still representative of the polysaccharide repeating unit and suitable to be used for biological activity tests. Acid generated oligosaccharides were separated by SEC on a Bio Gel P2 column and the obtained elution profiles of hydrolysates from PLS-B and PLS-C are reported in Figure 3. It can be appreciated that the two chromatograms are qualitatively very similar: the main difference is that the hydrolysate obtained from PLS-C has a more intense peak eluting at the void volume of the column (VoC) than that one generated from PLS-B (VoB). Peaks were named B-I to B-VI and C-I to C-VI; in both samples, the most retained peak was disregarded since from our previous work [32], it was known to contain salt and monosaccharides.

### 3.2. Structural Characterization of the Oligosaccharides Obtained after Mild Acid Hydrolysis of Tramesan^©^

In a previous publication [32], the characterization of oligosaccharides generated from Tramesan^©^ was carried out up to the tetrasaccharides. In the current study, larger oligosaccharides were needed for the biological tests and they were structurally characterized by ESI-MS and ^1^H NMR spectroscopy, taking advantage of the results already in our possession about di- tri- and tetrasaccharides.

**Figure 4:**
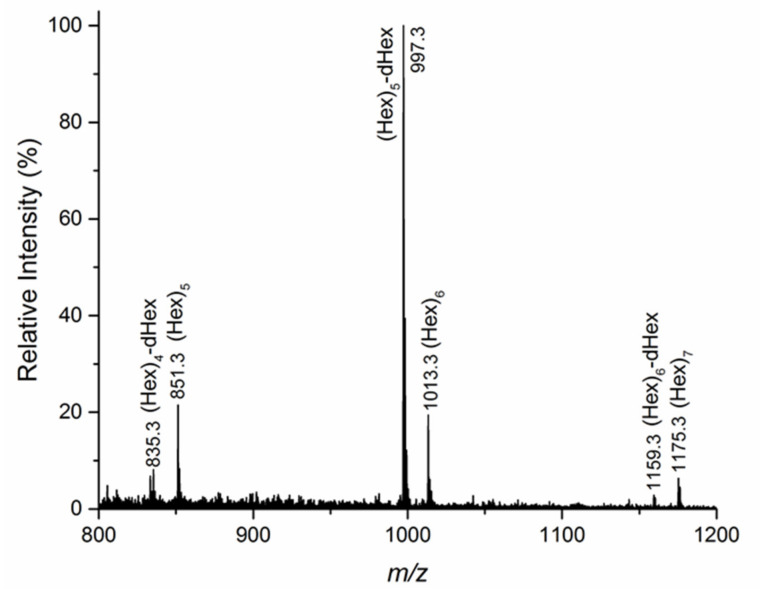
Electrospray ionization-mass spectrometry (ESI-MS) spectrum of sample B II obtained after hydrolysis of the polysaccharide purified from the *T. Versicolor* culture filtrate. The most abundant (M+Na)^+^ oligosaccharides’ parent ions are indicated.

Oligosaccharides BI-BVI and CI-CVI were first subjected to ESI-MS, which gave information on their size and composition in terms of hexoses (Hex) and deoxy-hexoses (dHex). MS^2^ of parent ions (data not shown) indicated that Fuc was always in a terminal position. As an example, the ESI-MS spectrum of BII is shown in Figure 4: all ions correspond to sodiated adducts. The parent ion at 997.3 u was assigned to a hexasaccharide composed of five Hex and one d-Hex residues, while the ion at 1103.3 u was assigned to a hexasaccharide containing only Hex residues. It can also be appreciated that BII contained small amounts of a pentasaccharide and a heptasaccharide, both consisting only of Hex residues. 

As expected from the very similar elution profiles (Figure 3), the size of oligosaccharides contained in BI–BVI peaks was identical to the respective CI–CVI ones, and in good agreement with previous findings [32]. The size and composition of the main oligosaccharides present in each peak are reported in Table 1. In the same fashion of BII, other peaks also contained small amounts of oligosaccharides composed of one more, or one less, Hex residue.

**Figure 5:**
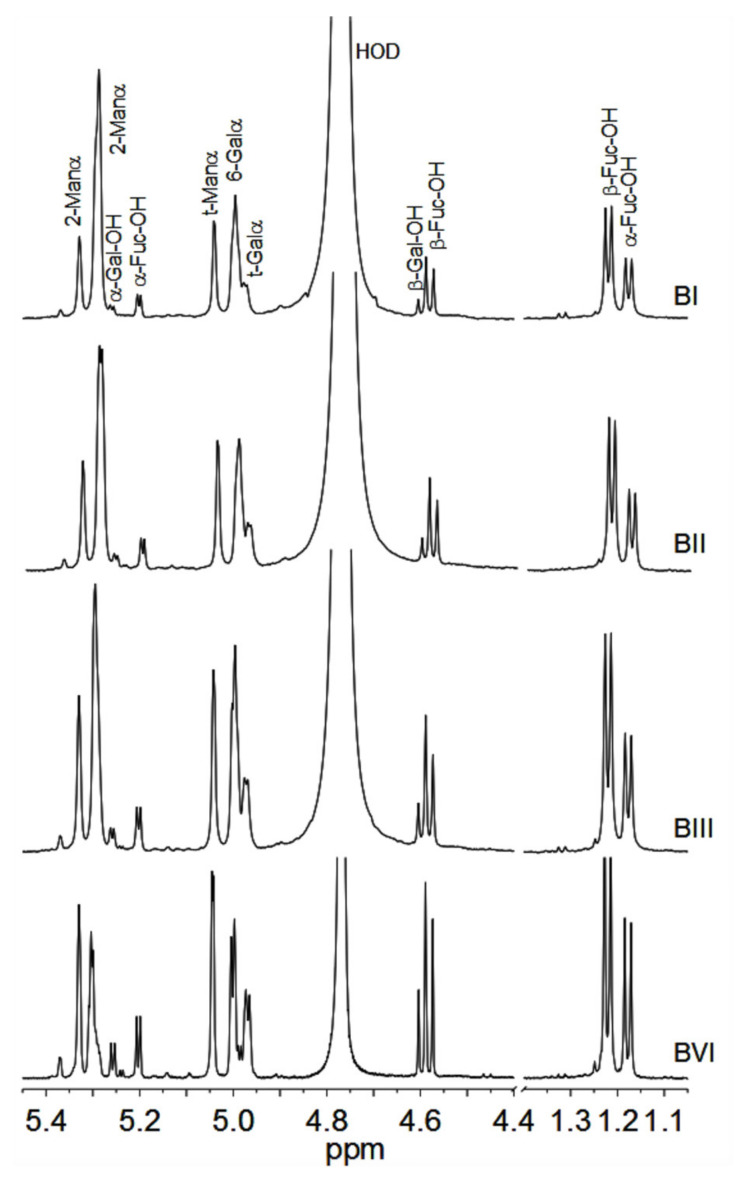
Anomeric and methyl regions of the ^1^H NMR spectra of samples BI, BII, BIII, and BIV. Assignment of main signals is shown.

After having determined the size of the oligosaccharides in each peak BI to BVI and CI to CVI, they were subjected to ^1^H NMR spectroscopy, which confirmed the identity of the B series with the C series of saccharides. Moreover, ^1^H NMR spectra of BVI and CVI showed that they contained mixtures of the disaccharides α-Man-Fuc-OH and α-Gal-Gal-OH, while BV and CV contained the trisaccharides (α-Man)_2_-Fuc-OH and (α-Gal)_2_-Gal-OH, in very good agreement with published assignments [39]. Identification of the oligosaccharides in BI–BVI and CI–CIV was made by comparison with the spectra of the smaller size oligosaccharides. The anomeric and methyl regions of the ^1^H NMR spectra of BI, BII, BIII, and BIV samples are reported in Figure 5, together with assignments of the main resonances. Detection of two doublets at about 1.24 ppm belonging to the C-6 methyl group of fucose is indicative of the α- and β-anomeric equilibrium of the reducing end residue. In the anomeric regions, the α-anomers of Fuc (α-Fuc-OH) and Gal (α-Gal-OH) reducing end resonate at 5.20 and 5.26 ppm, respectively, and the respective β-anomers at 4.58 (β-Fuc-OH) and 4.60 (β-Gal-OH) ppm. The presence of two reducing sugars confirmed that two structurally different oligosaccharides are present in the samples BI–BIV.

The occurrence H1 of linked Gal residues in the 5.02–4.95 ppm range indicated that they are in the α-anomeric configuration. ^1^J_C1-H1_ were detected in a coupled HSQC experiment and the constant values measured were in the interval 173–176 Hz thus establishing that all H1 of Man and Gal are in the α-anomeric configuration [39]. The signal integration values indicated that the oligosaccharides containing Man and Fuc were in higher amounts than those composed of Gal. The 2D NMR experiments were carried out on BIV sample in order to confirm the position of the glycosidic linkages. From the COSY plot (Figure S1) three cross peaks at 5.33–4.08 ppm, 5.30–4.10 ppm, and 5.04–4.06 ppm, which refer to the H1–H2 cross magnetization, are typical of Man residues, while the three cross peaks at 5.26–3.81 ppm, 5.00–3.83 ppm, and 4.97–3.82 ppm are attributable to Gal residues. Inspection of the HSQC spectrum (Figure S2) revealed the signals relative to carbon atoms involved in glycosidic linkages, which typically resonate at ppm higher than the respective unbound carbon atoms. In fact, C2 of the in chain mannose residues were found at 79.5 and 79.4 ppm, instead of about 70 ppm, and Gal C6 were located at 67 ppm instead of 62 ppm. Moreover, C3 of reducing Fuc residue was found at 78.4 ppm and 80.7 ppm, for the α- and β- anomers, respectively. In Table 2, a summary of the oligosaccharides present in each B and C fraction is reported.

### 3.3. AFB_1_ and OTA Inhibition Assay for the Oligosaccharides of Tramesan^©^

**Figure 6:**
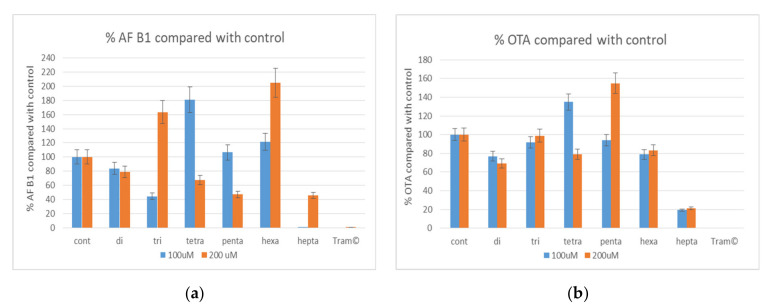
(**a**,**b**). Relative inhibition by different oligosaccharides and Tramesan^©^ of Aflatoxin B1 (**a**) and Ochratoxin A (**b**) production by *A. flavus* NRRL 3357 and *A. carbonarius* strain. Bars represent the mean ± SD of three determinations of five separate experiments. (cont—control; di—disaccharides; tri—trisaccharides; tetra—tetrasaccharides; penta—pentasaccharides; hexa—hexasaccharides; hepta—heptasaccharides; Tram—Tramesan^©^).

Each oligosaccharide fraction was tested for inhibition of mycotoxin production by *A. flavus* 3357 and *A. carbonarius* using a 96-well microtiter plate assay. Since oligosaccharide amounts were very low the concentration of 100 μM and 200 μM solutions of the oligosaccharides dissolved in water were used. Double concentrated potato dextrose broth (48 g/L), 100 μL of mixture (water solution of different oligosaccharides), and 10 μL of conidial suspension (1000 con/mL) were used for the experiment. The results, reported in Figure 6, showed that oligosaccharides longer than seven units showed the highest rate of mycotoxin inhibition, while the smaller ones had no significant inhibiting activity. In fact, some smaller saccharides stimulated mycotoxin production (tri, tetra and hexasaccharides AFB1 and tetra and pentasaccharides OTA). Moreover, the concentration differently affected inhibiting effects. In some cases (tri, hexa, and heptasaccharides for AFB1 and pentasaccharides for OTA) the lower saccharide concentration (100 μL) gave much higher inhibition than the higher one (200 μL).

## 4. Discussion

Polysaccharides derived from mushroom metabolites showed the potential to becoming a control agent for mycotoxin production [30,33,34,35,36]. One of the most promising biocontrol producers is the edible non-toxic basidiomycete *T. versicolor*. Previous studies indicated the potential activity of extracts from culture filtrate of mycelia of *T. versicolor* and its characterized polysaccharide Tramesan^©^, in controlling the growth and secondary metabolism (e.g., mycotoxins) of plant pathogenic fungi. Furthermore, studies have shown that the bioactivity of Tramesan^©^ relies mostly on its ability to act as a pro-antioxidant molecule, regardless of the biological system on which it was applied [30]. The structural investigation of Tramesan^©^ indicated that it contains stretches of 6-linked-Gal with Man side chains on Gal C2 glycoside bond. Although the positive properties of Tramesan^©^ have been proven, due to changing environmental conditions, random mutations may occur, which could alter the structure of the active polysaccharide moiety. The purified exopolysaccharide of *T. versicolor* was partially hydrolyzed and separated by size exclusion chromatography into oligosaccharide fractions ranging in size from two to seven units.

Each fraction was individually tested (100 µM and 200 µM concentrations) for AFB_1_ inhibition production by *A. flavus* and OTA control in *A. carbonarius*. Fragments smaller than seven units showed no significant effect on mycotoxin inhibition, whereas heptasaccharides showed inhibitory activity up to 90% (100 µM) and 55% (200 µM) in *A. flavus* and up to 81% (100 µM) and 78% (200 µM) in *A. carbonarius*. Since all of the oligosaccharides are composed of a mixture of two species of the same length but with different composition, at present it is not possible to determine which one, of the two fractions, is active, or if both have to be present. In fact, synergistic or antagonistic effects of the two different heptamers from the mixture on aflatoxin synthesis cannot be excluded. In future research, it will be necessary to separate the heptamers to characterize the exact structure of the active moiety and to avoid the potential interaction of two different heptamers. It is worth noting that for di-, three-, penta-, and hexasaccharides, a higher concentration of oligosaccharides has shown to promote aflatoxin synthesis. Moreover, the production of OTA was enhanced by the presence of tetramers and pentamers. This could be explained by the use of oligosaccharides as a carbohydrate supplementary source, which in turn favors mycotoxin synthesis [40]. Furthermore, the reported results indicate that a lower concentration of heptasaccharide is more efficient in inhibiting the synthesis of aflatoxins. This could be due to differences in the structure, and to the fact that at higher concentrations the oligosaccharides could degrade into smaller fragments, some of which may stimulate aflatoxin synthesis. Aflatoxin synthesis is highly influenced by the type and concentration of available carbon sources such as: certain types of sugars, fatty acids, sterols, and synthetic triglycerides [41,42,43,44,45]. For instance, Davis and collaborators [46] studied the aflatoxin production and growth of *Aspergillus parasiticus* using various carbon sources and concluded that, generally, compounds that are normally oxidized through both the hexose monophosphate and the glycolytic pathways, supported both growth and aflatoxin production. However, oligosaccharide concentration and aflatoxin inhibition are not always directly correlated, as evidenced in the results obtained using tetrasaccharide fractions, reported in Figure 6. This phenomenon was already reported in previous studies on mycotoxin inhibition [40]. In fact, these may be dose dependent actions, in which we have an increased effect of low concentration of sugar on aflatoxin synthesis. In addition, heptasaccharides, which are the largest fragments investigated, have shown the greatest influence on the control of mycotoxin synthesis. From these results, we can assume that the active fraction is greater than six units, and that a lower concentration of heptasaccharides has a better effect on aflatoxin and ochratoxin inhibition. These results are also consistent with previous research on the correlation between polysaccharide size and their activity in mycotoxin inhibition. Moreover, Scarpari and collaborators [14] proved that semi-purified culture filtrate of *T. versicolor* containing molecules with a supposed molecular mass > 3.0 kDa, was most effective in enhancing antioxidant activities and drastically inhibiting the biosynthesis of aflatoxins in *A. flavus*. Thus, we might assume that the size of the oligosaccharides is important for the binding to a specific receptor in the cell wall, and that longer oligosaccharides are more likely to bind and elicit a cell response. 

Similarly, chitin can prime defense in plants in oligosaccharidic form [47]. Notably, the “pace” provided by an heptamer/octamer of chitin is optimal to bind to the Pattern Related Receptors (PRR), CERK1/CEBiP (in a “sandwich” modality); and elicit the defense responses such as PTI (PAMP triggered immunity) [48]. This research indicates some novel insights on the active structures of Tramesan^©^ oligosaccharides, which could result in understanding the mode-of-action of Tramesan^©^ and shedding light into the way in which fungi can perceive signals deriving from other fungi and shape their lifestyles accordingly. Intriguingly, Tramesan^©^ has no obvious antifungal or phytotoxic effects [30,49]. These evidences can support the hypothesis that Tramesan^®^ may display a biological effect on the basis of its “signal” more than “toxic” nature. 

Moreover, the production via synthesis of the active moiety of Tramesan^©^ could facilitate its widespread use in agriculture. In conclusion, the active part of the *T. versicolor* polysaccharide could be a possible new eco-friendly tool for mycotoxin control.

## Figures and Tables

**Table 1:** Size and composition of the oligosaccharides present in the Tramesan^©^ fractions after acidic hydrolysis of the native polymer.

Tramesan Fractions	Size	Composition of Oligosaccharides
BVI and CVI	disaccharides	Hex-dHex and (Hex)2
BV and CV	trisaccharides	(Hex)2-dHex and (Hex)_3_
BIV and CIV	tetrasccharides	(Hex)_3_-dHex and (Hex)_4_
BIII and CIII	pentasaccharides	(Hex)_4_-dHex and (Hex)_5_
BII and CII	hexasaccharides	(Hex)_5_-dHex and (Hex)_6_
BI and CI	heptasaccharides	(Hex)_6_-dHex and (Hex)_7_

**Table 2:** Oligosaccharides present in each B and C fraction of the hydrolyzed Tramesan^©^.

Tramesan Fractions	Size	Composition of Oligosaccharides
BVI and CVI	disaccharides	αMan-Fuc-OH and αGal-Gal-OH
BV and CV	trisaccharides	(αMan)2-Fuc-OH and (αGal)2-Gal-OH
BIV and CIV	tetrasccharides	(αMan)_3_-Fuc-OH and (αGal)_3_-Gal-OH
BIII and CIII	pentasaccharides	(αMan)_4_-Fuc-OH and (αGal)_4_-Gal-OH
BII and CII	hexasaccharides	(αMan)_5_-Fuc-OH and (αGal)_5_-Gal-OH
BI and CI	heptasaccharides	(αMan)_6_-Fuc-OH and (αGal)_6_-Gal-OH

## Data Availability

All results are contained within the article.

## Supplementary Materials

The following are available online at https://www.mdpi.com/2218-273X/11/2/243/s1, Figure S1: COSY, Figure S2: HSQC.
