# Supplementary material for: Oligosaccharides Derived from Tramesan: Their Structure and Activity on Mycotoxin Inhibition in Aspergillus flavus and Aspergillus carbonarius
Source: Biomolecules. 2021 Feb 8;11(2):243. doi: 10.3390/biom11020243 (PMC7914814; doi:10.3390/biom11020243)
Supplement: Supplementary file 1 [file biomolecules-11-00243-s001.pdf]

Oligosaccharides Derived from Tramesan: Their Structure and Activity on Mycotoxin Inhibition in *Aspergillus flavus* and *Aspergillus carbonarius*

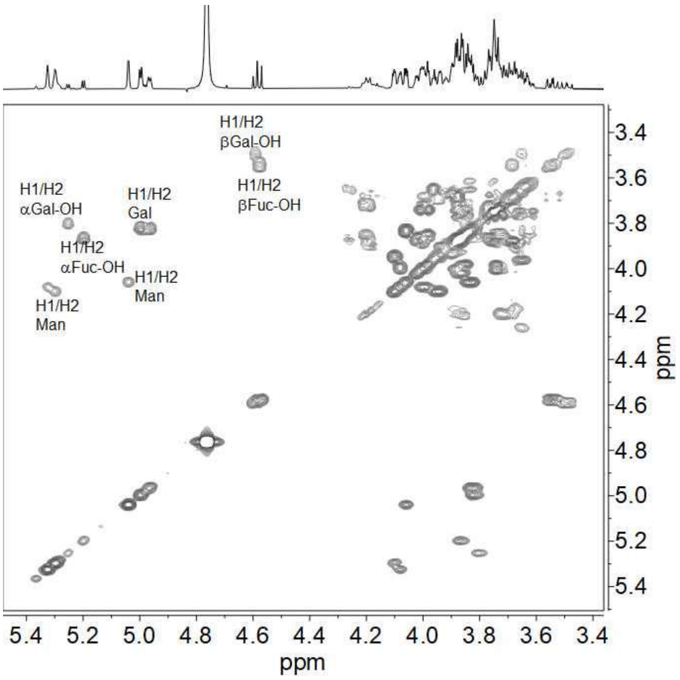

Figure S1: COSY

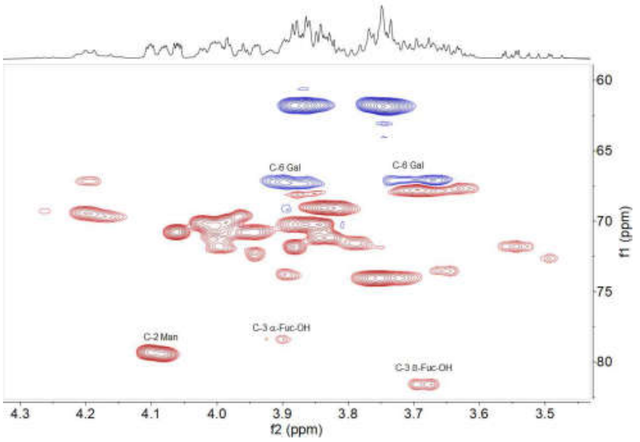

Figure S2: HSQC
